# HIV/AIDS-Associated Knowledge and Attitudes towards Treating Disadvantaged Communities among Pre-Community-Based Dental Education Dental Students in the U.S.

**DOI:** 10.3390/ijerph21070927

**Published:** 2024-07-16

**Authors:** Aditi Tomar, Hannah Balcezak, Shirley Lewis Miranda, Marie C. Latortue, Richa Chinchkhandi, Lisa Wigfall

**Affiliations:** 1 The University of North Carolina at Chapel Hill, Chapel Hill, NC 27599, USA; 2 UTHealth Houston School of Dentistry, Houston, TX 77054, USA; hannahbalcezak@tamu.edu (H.B.); marie.c.latortue@uth.tmc.edu (M.C.L.); 3 Texas A&M School of Dentistry, Dallas, TX 75246, USA; slewis@tamu.edu; 4 Texas A&M School of Public Health, College Station, TX 77843, USA; richachin1@tamu.edu; 5 National Institutes of Health, Bethesda, MD 20892, USA; lisa.wigfall@nih.gov

**Keywords:** HIV/AIDS, community-based dental education (CBDE), people living with HIV/AIDS (PLWHA)

## Abstract

This study examines HIV/AIDS-associated knowledge and attitudes towards treating disadvantaged communities among predoctoral dental students in U.S. dental schools who have not yet commenced their Community-Based Dental Education (CBDE) training. It also compares the difference in the knowledge and attitudes between students with reported community engagement with PLWHA and those without. Our study comprised 89 predoctoral dental students within their D1–D3 years of dental school who had not yet initiated their Community-Based Dental Education (referred to as pre-CBDE dental students). Their responses were collected via an online survey. The participants were 68% females, 94% heterosexual, and with a mean age (years) of 25.9 ± 3.5 SD. The majority (62%) were in their first (D1) and second (D2) years of dental education. Of the thirty knowledge questions, only five received a minimum of 90% correct responses. Similarly, we found no statistical differences in most of the knowledge/attitude sections between those with and without prior PLWHA exposure. Encouragingly, 90% of our participants reported prior experience working with disadvantaged communities. Early community engagement fosters a sense of professional responsibility towards administering dental care to disadvantaged communities and we propose that it must be encouraged among aspiring dental school students.

## 1. Introduction

Inadequate access to oral health services remains a persistent challenge, disproportionately affecting disadvantaged groups and worsening their overall quality of life [1]. Despite significant progress in the diagnosis and treatment of oral diseases, specific communities including racial/ethnic minorities, those on a low income, People Living with HIV/AIDS (PLWHA), people who inject drugs, and homeless people continue to experience high and unacceptable burdens of oral health challenges [2,3,4]. Therefore, it is crucial to ensure sufficient and prompt access to oral health care for these individuals.

Students enrolled in dental schools represent a robust, dynamic group of individuals, capable of improving the existing scenario related to HIV/AIDS care delivery in dental settings. As future oral health professionals, they are not only expected to adhere to high moral and ethical standards in providing optimal treatment and care to disadvantaged groups, but also contribute to a stigma-free, non-judgmental environment for oral health care [5]. To instill a sense of community service among dental students, the U.S. Health Resources and Services Administration HIV/AIDS Bureau’s Community-Based Dental Partnership Program (CBDPP) recommends that U.S.-based dental schools implement the Community-Based Dental Education (CBDE) program as a part of the curriculum [6]. CBDE trains dental students to provide care to underserved communities, through a combination of didactic and clinical work in community-based dental clinics, frequently visited by disadvantaged groups at an elevated risk of HIV [6,7]. The objective of CBDE is to provide early exposure to community dental practice, cultivating a proactive readiness to serve PLWHA in dental practice.

The knowledge, attitudes, and intentions of oral health care providers play a crucial role in shaping the quality of oral care provided to PLWHA [7]. Previous studies have shown that dental professionals may exhibit limited knowledge regarding HIV/AIDS diagnosis, contagion, and treatment guidelines and infection control practices [8]. This knowledge gap often amplifies the prevailing stigma associated with treating HIV patients in dental practice, preventing People Living with HIV (PLWHA) from accessing quality dental care [9,10,11].

Early exposure to underserved populations has been shown to be positively associated with the willingness to treat this population in dental careers. Studies show that personal experiences with friends and family on Medicaid before entering dental school, as well as experiences during dental school, can have a positive effect on how comfortable dentists are when treating underserved patients [12]. As part of their admission process, many dental schools strongly prioritize applicants with prior shadowing experience in dental practices [13]. The American Dental Education Association (ADEA) specifically encourages prospective applicants to engage in shadowing at community and federal clinics that cater to low-income populations [13]. Besides enhancing their chances of admission, shadowing at community clinics also offers a comprehensive insight into the dental management of underserved groups, including individuals living with HIV (PLWHA).

While a few studies have reported the success of community CBDE and similar training programs in improving HIV-related knowledge and attitudes among dental students [6,7], there is a need for a more in-depth understanding about the broad spectrum of PLWHA/HIV-related knowledge and attitudes specifically before the initiation of CBDE—a gap our study aims to address. Such a comprehensive understanding will pinpoint specific areas where pre-CBDE dental students may lack HIV-related knowledge or hold certain attitudes towards the dental management of HIV patients. Subsequently, the CBDE training could be tailored to address these specific areas.

Our study also aims to investigate whether prior experience working with PLWHA affects HIV-related knowledge and attitudes toward treating PLWHA. Therefore, our study aims are twofold: (1) Explore knowledge regarding HIV/AIDS-associated transmission/screening and oral manifestation; attitudes towards treating PLWHA, and anticipatory attitudes towards administering oral care to disadvantaged community groups, namely, people on a low income, people who inject drugs, homeless people, and PLWHA, among U.S.-based pre-CBDE dental school students. (2) Compare the difference in knowledge and attitudes between pre-CBDE dental students with reported experience working with PLWHA versus pre-CBDE dental students without reported experience working with PLWHA. For the purposes of this manuscript, we will refer to the predoctoral dental students in our study who have not yet begun their dental training as pre-CBDE dental students.

## 2. Methods

### 2.1. Sample

This study was approved by the University’s Institutional Review Board and involved a purposive sample of dental students from thirteen U.S.-based dental schools. In January 2019, the principal investigator (LTW) sent a recruitment email to the Academic Deans of Research and/or Student Affairs at the 66 U.S.-based schools/colleges of dentistry, out of whom 2 declined, 51 did not respond, and 13 agreed to participate in the study. The 13 schools are located in the U.S. Census region states, as follows: Northeast: New York; West: Arizona, California; Midwest: Missouri, Ohio; South: Florida, Kentucky, North Carolina, Tennessee, Texas, and West Virginia. Next, the survey was distributed among 1st to 4th year (D1–D4) predoctoral dental students. Eligibility criteria for participation included predoctoral dental students enrolled in the DDS/DMD program, who had not commenced CBDE at the time of the study. Data were collected over a three-month period between January 2019 and March 2019. Our final sample comprised 89 pre-CBDE, predoctoral dental students in the U.S.

### 2.2. Survey

A 30 min self-administered Qualtrics survey was employed for data collection. The questionnaire comprised the following parts: a four-item demographics section, a four-item section capturing prior experience working with disadvantaged communities, a fifteen-item section on evaluating knowledge regarding HIV/AIDS transmission and diagnosis, a fifteen-item section on knowledge regarding oral manifestation of AIDS, a seventeen-item section assessing attitude regarding treatment and interaction with PLWHA, and lastly, an eight-item section assessing attitude (willingness) towards administering dental care to disadvantaged communities during CBDE and after graduation. Our survey instrument was drawn from surveys used in previous studies [7,14,15]. However, e excluded a few attitude items from our final analysis. In accordance with the study conducted by Kumar and colleagues [5], the statement “Dentists with HIV/AIDS should not be allowed to treat patients” was not used for our analysis, as there is still no definite consensus on whether HIV-positive health personnel should treat patients. Similarly, we excluded the statement “A blood test should be taken for diagnosis of HIV infection in all dental patients”, since it is limited by the clause that supports patients’ free will to refuse HIV testing.

The two knowledge sections recorded participant responses as “yes/no” and “true/false”. Correct knowledge responses were coded as 1, and incorrect responses were coded as 0. Attitude questions were assessed on a five-point Likert scale (strongly agree, agree, neutral, disagree, and strongly disagree). For questions implicating negative attitudes, a value of 5 corresponded with “strongly disagree”, 4 was given to “disagree”, 3 for “neutral”, 2 for “agree”, and 1 for “strongly agree”. On the other hand, for questions implicating positive attitudes, a value of 5 corresponded with “strongly agree”, 4 was given to “agree”, 3 for “neutral”, 2 for “disagree”, and 1 for “strongly disagree”.

### 2.3. Data Analysis

A total of 149 participants from the thirteen participating schools responded to the study questionnaire, of whom 117 answered more than 50% of the applicable questions. Since we do not have a record of the total number of students who received the survey and are unable to retrospectively obtain the information, the survey response rate cannot be calculated. The participants (n = 28) who responded “yes” to “Have you begun Community-Based Dental Education” were excluded from the analysis for this paper, yielding a final sample size of 89 dental students. Data analysis was performed using Stata/SE 16.0. Cross-tabulation was used to calculate the percentages and frequencies for both demographic, knowledge, and attitude variables. Pearson’s chi-square was used to determine whether the demographic, knowledge, and attitude/belief responses between students with and without prior experience with PLWHA were significantly different. A *p*-value < 0.05 was considered statistically significant. Student’s *t*-test was used to compare the significant difference in mean knowledge and attitude scores between students with and without pre-CBDE experience working with PLWHA.

## 3. Results

Table 1 illustrates the sample characteristics of the participants. Of the total 89 participants, 68% were females, 94% heterosexual, and with a mean age (years) of 25.9 ± 3.5 *SD*. Around one-third (31.5%) was in the first (D1) year of the DDS/DMD program, 30.3% were D2 (second year students), and 38.2% were D3 (third year) students. The majority (93.2%) were members of the American Dental Association (ADA). An overwhelming majority (90%) reported prior experience working with the following disadvantaged groups: low-income communities (90%), people who inject drugs (43%), homeless people (39.3%), PLWHA (40.4%). Statistically significant differences were noted in the demographic characteristics, specifically in terms of age (*p* = 0.00) and year of study (*p* = 0.00), when comparing students with experience working with PLWHA and those without experience. Interestingly, the percentage of students who had worked with low-income communities (100%, *p* = 0.00), people who inject drugs (61%, *p* = 0.00) and homeless people (56%, *p* = 0.01) was significantly higher among those who indicated prior experience with PLWHA compared to those without.

Table 2 describes the knowledge responses related to HIV/AIDS transmission and screening. Interestingly, there were no significant differences in any of the individual knowledge items between the students with and without experience with PLWHA. The proportion of students who provided correct responses ranged between 41.5% and 95.5%. Another surprising observation was that only one-third (5/15) of the questions had at least 90% correct responses. Having previous experience working with People Living with HIV/AIDS (PLWHA) had a statistically significant impact on the mean knowledge scores for this section (*p* > 0.05).

Table 3 depicts the knowledge responses concerning the oral clinical manifestations of AIDS. Unfortunately, none of the questions received at least 90% correct responses, indicating less favorable outcomes in this particular section. The proportion of participants who responded correctly ranged between 40.9% and 88.8%. The majority were aware of the frequently encountered clinical manifestations of AIDS, such as oral candidiasis (88.8%) and Kaposi’s sarcoma (85.4%). Similar to the previous knowledge section, prior experience working with PLWHA showed no significant impact on the mean knowledge scores for this section (*p* > 0.05).

The attitudes surrounding the dental management of PLWHA are presented in Table 4. The majority of the participants exhibited positive attitudes toward providing treatment and care to clients who are HIV-positive. Overall, 92.2% strongly disagreed/disagreed that treating HIV/AIDS patients means wasting national resources, 91% thought they were morally responsible for treating HIV-positive patients, and 91% indicated a willingness (strongly agree/agreed) to treat PLWHA. The majority (92%) rightly acknowledged that all dental patients should be considered to be potentially infectious for HIV. Eighty percent of the participants expressed a willingness (strongly agree/agree) to administer CPR to an HIV-positive client. However, the patterns differed between those with and without prior experience with PLWHA, with the majority (94.3%) of the participants with prior PLWHA work experience demonstrating a significantly higher willingness (strongly agree/agree) to perform CPR compared to only 51.4% of those who had not worked with PLWHA (*p* = 0.00). Among the participants with prior experience working with PLWHA, half (54%) strongly agreed that their infection control knowledge was enough to treat HIV/AIDS clients, compared to a significantly lower proportion of 19% of participants without PLWHA experience (*p* < 0.01). No significant differences were observed in the mean attitude scores between the two participant groups.

Table 5 describes the participants’ anticipatory willingness towards treating disadvantaged population groups during their CBDE training. Overall, 75% expressed a willingness (somewhat–extremely comfortable) to treat the homeless, 68% indicated a willingness to treat PLWHA, and 63% reported a willingness to provide dental care to people who inject drugs. A substantial proportion (90%) indicated a willingness to provide care to low-income communities. Compared to the participants without experience with PLWHA, those with PLWHA experience were willing to treat PLWHA (60% vs. 78%, *p* = 0.01) and homeless communities (70% vs.83%, *p* = 0.01). The overall mean attitude score for this section was 15.73 ± 4.06 SD, with a range of 0–20. Expectedly, the participants who indicated prior PLWHA experience had a significantly higher mean attitude score compared to the ones without reported PLWHA exposure (17.05 ± 2.8 SD) vs.14.80 ± 4.5 SD, *p* = 0.01).

Lastly, the anticipatory willingness to treat disadvantaged population groups after graduation has been outlined in Table 6. The participants overwhelmingly expressed a strong willingness to treat disadvantaged communities after graduating from dental school: 98.9% for low-income populations, 97.7% for homeless people, 92.1% for people who inject drugs, and 95.5% for People Living with HIV/AIDS. No significant differences were observed in any of the individual attitude items between the participants with and without PLWHA experience. The mean favorable attitude score was recorded as 3.84 ± 0.58 SD (score range 0–4), with no significant (*p* > 0.05) differences between the two participant groups.

## 4. Discussion

Our study investigates the knowledge regarding HIV/AIDS-related transmission, screening, and oral manifestations and attitudes towards managing disadvantaged population groups (during CBDE and after graduation) among U.S.-based predoctoral dental school students who have not commenced their CBDE training. We compared the differences in knowledge and attitudes between the students with previous exposure to PLWHA and those without exposure to PLWHA.

Encouragingly, the participants in our study demonstrated positive responses regarding their knowledge about transmission and infection control. Specifically, 93% agreed/strongly agreed that PLWHA can infect dental workers, 94% reported needle stick injury may be a potential route for transmission, 82% dental workers said saliva can act as an intermediary for HIV transmission. This is slightly more promising than the study by Hamershock and colleagues, where 77.4% of pre-CBDE dental students agreed/strongly agreed that needle stick injury may be a potential route for transmission, and 78.4% recognized saliva as a potential transmission route among predoctoral dental students.

Most (90%) of the students in our study reported previous experience working with at least one of the following disadvantaged groups: people on a low income, PLWHA, people who inject drugs, and homeless people, implicating that future dental professionals are motivated to serve disadvantaged communities. This finding is noteworthy and demonstrates a spirit of social responsibility among future dental professionals. Additionally, as discussed in other studies, early community engagement is essential to acquaint aspiring dental professionals with clinical adeptness and an empathic approach to managing disadvantaged groups [7,8].

Interestingly, there were no significant differences observed in the mean scores pertaining to four out of the five knowledge and attitude sections between the students who indicated previous experience working with PLWHA and students without previous exposure. The only exception is the section assessing attitudes towards treating disadvantaged groups during CBDE, where students with prior PLWHA exposure exhibited more favorable attitudes toward treating PLWHA and homeless individuals compared to those without PLWHA exposure. However, in terms of providing dental services to PLWHA after graduation, there was lack of significance between those with and those without PLWHA exposure and both demonstrated an equal enthusiasm to treat disadvantaged groups. The existing literature does not provide a comparison of the attitudes towards offering dental care to disadvantaged groups based on prior exposure to PLWHA. Due to this gap, we cannot determine how exposure to PLWHA may impact attitudes toward offering care to disadvantaged groups during CBDE, but not after graduation. Nevertheless, it is important to highlight that upon graduation, both groups exhibit equal motivation to offer dental services to disadvantaged groups. Therefore, dental school applicants should continue engaging in community dental service, fostering valuable experience in addressing the specific clinical challenges associated with managing disadvantaged communities.

In the total sample, only five of the thirty knowledge entities had at least 90% correct responses. This finding may be linked to the disproportionate (62%) majority of D1 and D2 dental students in our study. Students in their initial years may have minimal curricular exposure to HIV/AIDS-related topics and may therefore be less informed about managing PLWHA in dental settings. Lastly, it must be noted that many studies both within [6,7] and outside the U.S. [5,14,15,16] have evaluated knowledge regarding HIV/AIDS-related transmission, screening, and oral manifestations and attitudes towards managing disadvantaged population groups. However, to our knowledge, this is the first study where, besides an in-depth assessment of knowledge and attitudes, insight into the status of previous experiences with disadvantaged groups is provided. We have also depicted how prior experience working with PLWHA may potentially influence HIV/AIDS-related knowledge and attitudes towards the dental management of certain disadvantaged groups. By focusing on students who have not officially commenced their CBDE training, this study offers a distinctive perspective on the potential impact of early exposure and experiences working with PLWHA on their willingness to treat PLWHA and other disadvantaged groups.

## 5. Strengths and Limitations

There are some noted weaknesses of our study. First, we did not capture the exact timepoints of procuring prior PLWHA experience, that is, when exactly the participant engaged with PLWHA. Second, we did not inquire about the source (community dental clinic/voluntary social work/research experience) of acquiring prior experience working with PLWHA. Thirdly, our study lacks a response rate calculation, which has been addressed in the Methods Section. Lastly, our sample size of 89 students, while representing various regions of the U.S., is relatively small. Our findings, therefore, may not be generalizable across all dental schools in the U.S. Additionally, the small sample size may also have limited the statistical power to adequately detect differences between pre-CBDE students with and without experience working with PLWHA. Lastly, as previously mentioned, the survey items were derived from previous studies that measured HIV-related knowledge and attitudes among dental students, rather than being developed firsthand. Our future steps will involve conducting the study with a larger group and developing a reliable and validated survey instrument tailored specifically to pre-CBDE dental students.

A notable strength of our study is that at least one school was represented for each US Census region. Additionally, this is the first study that investigates HIV/AIDS-associated knowledge, attitudes, and anticipatory willingness towards treating disadvantaged communities among dental school students who have not commenced their CBDE training. This underscores the importance of consistently encouraging students to participate in service-oriented activities even before entering dental schools.

## 6. Conclusions

The results from this study underscore the persistent gap in PLWHA-related knowledge and attitudes among dental students. Interestingly, there is no significant association observed between prior experience working with PLWHA, HIV/AIDS-related knowledge, and anticipatory attitudes towards treating disadvantaged communities. However, we need to explore if previous experience working with any of the other disadvantaged community groups may suggest otherwise. Therefore, as our next steps, we will broaden our research scope to investigate whether working with disadvantaged community groups (people on a low income/people who inject drugs/homeless) other than PLWHA may have an impact on HIV/AIDS-related knowledge/anticipatory attitudes among pre-CBDE dental students.

## Figures and Tables

**Table 1:** Sample characteristics.

Characteristic	Total N = 89 N (%)	Pre-CBDE Dental Students with Previous Community Experience Working with PLWH N = 36 N (%)	Pre-CBDE Dental Students without Previous Experience Working with PLWH N = 53 N (%)	*p*-Value
**Mean age**	83 (25.9 ± 3.5 SD)	32 (27.3 ± 4.6 SD)	51 (25.1 ± 2.4 SD)	0.00
**Year of study (N = 89)**				
D1	28 (31.5)	7 (19.4)	21 (39.6)	0.00
D2	27 (30.3)	8 (22.3)	19 (35.9)	
D3	34 (38.2)	21 (58.3)	13 (24.5)	
**Sex (N = 88)**				
Male	28 (31.8)	15 (41.7)	13 (25.0)	0.09
Female	60 (68.2)	21 (58.3)	39 (75.0)	
**Sexual orientation (N = 88)**				
Heterosexual	83 (94.3)	32 (88.9)	51 (98.1)	0.15
Gay	2 (2.3)	2 (5.5)	1 (1.9)	
Bisexual	1 (1.1)	1 (2.8)	0 (0.0)	
Something else	2 (2.3)	1 (2.8)	0 (0.0)	
**American Dental Association membership (N = 88)**				
Yes	82 (93.2)	34 (94.4)	48 (92.3)	0.69
No	6 (6.8)	2 (5.6)	4 (7.7)	
**Experience working with disadvantaged groups (N = 89)**				
People with low income				
Yes	80 (89.9)	36 (100.0)	44 (83.0)	0.00
No	9 (10.1)	0 (0.0)	9 (17.0)	
People who inject drugs				
Yes	38 (42.7)	22 (61.1)	16 (30.2)	0.00
No	51 (57.3)	14 (38.9)	37 (69.8)	
Homeless people				
Yes	35 (39.3)	20 (55.6)	15 (28.3)	0.01
No	54 (60.7)	16 (44.4)	38 (71.7)	

**Table 2:** Knowledge about HIV/AIDS transmission and screening.

Knowledge Question (True/False)	Total, N = 89 N (%)		Pre-CBDE Dental Students with Previous Community Experience Working with PLWH N = 36 N (%)		Pre-CBDE Dental Students without Previous Experience Working with PLWH N = 53 N (%)		*p*-Value
	Correct Response	Incorrect Response	Correct Response	Incorrect Response	Correct Response	Incorrect Response	
HIV/AIDS patients can contaminate dental workers	83 (93.3)	6 (6.7)	33 (91.7)	3 (8.3)	50 (94.3)	3 (5.7)	0.60
HIV/AIDS patients can be diagnosed with oral manifestations	78 (87.6)	11 (12.4)	31 (86.1)	5 (13.9)	47 (88.7)	6 (11.3)	0.71
ELISA is a screening test for HIV infection	75 (84.3)	14 (15.7)	28 (77.8)	8 (22.2)	47 (88.7)	6 (11.3)	0.16
Western blot is a definite test for HIV infection	56 (62.9)	33 (37.1)	20 (55.6)	16 (44.4)	36 (67.9)	17 (32.1)	0.24
Needle stick injury during dental treatment can transmit HIV	84 (94.4)	5 (5.6)	33 (91.7)	3 (8.3)	51 (96.2)	2 (3.8)	0.36
Dental workers can act as an intermediary for transmission of HIV	73 (82.0)	16 (18.0)	30 (83.3)	6 (16.7)	43 (81.1)	10 (18.9)	0.79
Saliva can be a vehicle for the transmission of AIDS ^a^	73 (82.0)	16(18.0)	32 (88.9)	4 (11.1)	41 (77.4)	12 (22.6)	0.16
Hepatitis B is more communicable than HIV/AIDS	78 (87.6)	11 (12.4)	33 (91.7)	3 (8.3)	45 (85.9)	8 (15.1)	0.34
All sterilization methods have cidal effects against HIV	59 (66.3)	30 (33.7)	23 (63.8)	13 (36.1)	36 (67.9)	17 (32.1)	0.69
Negative HIV tests surely indicate that the persons are free of viruses ^a^	81 (91.0)	8 (9.0)	31 (86.1)	5 (13.9)	50 (94.3)	3 (5.7)	0.18
There is a lot of HIV in the saliva of HIV/AIDS patients ^a^	85 (95.5)	4 (4.5)	35 (97.2)	1(2.78)	50 (94.3)	3 (5.7)	0.52
There are special dental clinics for treatment of AIDS patients in India ^a^	28 (41.5)	61 (68.5)	10 (27.8)	26 (72.2)	18 (34.0)	35 (66.0)	0.53
Presently, AIDS is the most important health problem in the world ^a^	69 (77.5)	20 (22.5)	30 (83.3)	6 (16.7)	39 (73.6)	14 (26.4)	0.28
CPR for patients with AIDS can transmit HIV infection ^a^	81 (91.0)	8 (9.0)	31 (86.1)	5 (13.9)	50 (94.3)	3 (5.7)	0.18
Mean knowledge score (Total possible score = 15)	12.17 ± 1.51 SD		12.02 ± 1.48 SD		12.28 ± 1.53 SD		0.44

Note. False = correct for ^a^ response.

**Table 3:** Oral manifestations of HIV/AIDS.

Knowledge Question (Yes/No)	Total N = 89 N (%)		Pre-CBDE Dental Students with Previous Community Experience Working with PLWH N = 36 N (%)		Students without Previous Experience Working with PLWH N = 53 N (%)		*p*-Value
	Correct Response	Incorrect Response	Correct Response	Incorrect Response	Correct Response	Incorrect Response	
Oral candidiasis	79 (88.8)	10 (11.2)	33 (91.7)	3 (8.3)	46 (86.8)	7 (13.2)	0.47
Kaposi’s sarcoma	76 (85.4)	13 (14.6)	28 (77.8)	8 (22.2)	48 (90.6)	4 (9.4)	0.09
Acute necrotizing ulcerative gingivitis (ANUG)	50 (56.2)	39 (43.8)	23 (63.9)	13 (36.1)	27 (51.0)	26 (49.0)	0.21
Major aphthous	46 (51.7)	43 (48.3)	19 (52.8)	17 (47.2)	27 (50.9)	26 (49.1)	0.91
Cytomegalovirus	45 (50.56)	44 (49.4)	20 (55.6)	16 (44.4)	24 (47.2)	28 (52.8)	0.44
Crohn’s disease	76 (85.4)	13 (14.6)	22 (88.9)	4 (11.1)	44 (83.0)	9 (17.0)	0.59
Hairy leukoplakia	59 (66.3)	30 (33.7)	21 (58.3)	15 (41.7)	38 (71.7)	15 (28.3)	0.26
Severe periodontitis	64 (71.9)	25 (28.1)	25 (69.4)	11 (30.6)	39 (78.6)	14 (26.4)	0.71
Xerostomia	58 (65.2)	31 (34.8)	21 (58.3)	15 (41.7)	37 (69.8)	16 (30.2)	0.20
Salivary gland infection	52 (58.4)	37 (41.6)	23 (63.9)	13 (36.1)	29 (54.7)	24 (45.3)	0.42
Gingivitis	54 (60.7)	35 (39.3)	23 (63.9)	12 (36.1)	31 (58.5)	22 (41.5)	0.70
Herpes Zoster	40 (40.9)	49 (55.1)	15 (41.7)	21 (58.3)	25 (47.2)	28 (52.8)	0.64
Herpes simplex	43 (48.3)	46 (51.7)	16 (44.4)	20 (55.6)	25 (50.9)	26 (49.1)	0.52
Condyloma	42 (47.2)	47 (52.8)	16 (44.4)	20 (55.6)	26 (49.1)	27 (50.9)	0.69
Papilloma	51 (57.30)	38 (42.7)	20 (55.6)	16 (44.4)	31 (58.5)	22 (41.5)	0.88
Mean knowledge score (Total possible score = 15)	9.38 ± 3.50 SD		9.30 ± 3.65 SD		9.43 ± 3.42 SD		*t*-test 0.43

**Table 4:** Attitude towards dental management of PLWH.

Characteristic	Total, N = 89 N (%)			Pre-CBDE Dental Students with Previous Community Experience Working with PLWH N = 36 N (%)			Pre-CBDE Dental Students without Previous Community Experience Working with PLWH N = 53 N (%)			*p*-Value
	Strongly Agree/ Agree	Neutral	Strongly Disagree/ Disagree	Strongly Agree/ Agree	Neutral	Strongly Disagree/ Disagree	Strongly Agree/ Agree	Neutral	Strongly Disagree/ Disagree	
Treatment of HIV/AIDS patients means wasting national resources ^a^	1 (1.1)	6 (6.7)	82 (92.2)	0 (0.0)	3 (8.3)	33 (91.7)	4 (7.6)	0 (0.0)	49 (92.4)	0.56
All dental patients should be considered potentially infectious for HIV	82 (92.1)	3 (3.4)	4 (4.5)	32 (88.9)	1 (2.7)	2 (8.4)	50 (94.2)	2 (3.8)	1 (1.9)	0.63
If I know that my friend has HIV, I end the friendship ^a^	2 (2.2)	20 (22.4)	81 (91)	0 (2.8)	3 (8.3)	32 (88.9)	1 (1.9)	3 (5.7)	49 (92.5)	0.64
Supporting HIV/AIDS patients improves community health	60 (68.2)	21 (23.9)	7 (7.9)	24 (68.6)	8 (22.9)	3 (8.6)	36 (67.9)	13 (30.1)	4 (7.5)	0.80
HIV/AIDS patients should be treated at a separate ward ^a^	5 (5.7)	14 (15.9)	69 (78.4)	2 (5.7)	5 (14.3)	28 (80.0)	3 (5.7)	9 (16.9)	41 (77.4)	0.45
I am morally responsible for treating HIV/AIDS patients	80 (91.0)	7 (7.9)	1 (1.1)	34 (97.2)	1 (2.9)	0 (0.0)	46 (86.8)	7 (13.2)	1 (1.9)	0.17
HIV/AIDS patients can live with others in the same place	83 (94.3)	5 (5.7)	0 (0.0)	33 (94.3)	2 (5.7)	0 (0.0)	50 (94.3)	3 (5.7)	0 (0.0)	0.71
I am not obligated to treat HIV/AIDS patients ^a^	11 (12.5)	14 (15.9)	63 (71.6)	5 (14.3)	4 (11.4)	26 (74.3)	6 (11.3)	10 (18.9)	37 (69.9)	0.44
HIV/AIDS patients can lead a normal life	79 (89.8)	7 (8.0)	2 (2.2)	32 (94.1)	2 (5.9)	0 (0.0)	47 (87.0)	5 (9.3)	2 (3.7)	0.51
I can safely treat HIV/AIDS patients	77 (87.5)	11 (12.5)	0 (0.0)	25 (71.4)	5 (14.3)	5 (14.3)	47 (88.6)	6 (11.4)	0 (0.0)	0.06
I will treat HIV/AIDS patients	80 (90.9)	8 (9.1)	0 (0.0)	33 (94.0)	2 (5.7)	0 (0.0)	47 (88.7)	6 (11.3)	0 (0.0)	0.09
My knowledge about infection control is enough to treat HIV/AIDS patients	62 (71)	14 (15.9)	12 (13.6)	27 (77.2)	5 (14.3)	3 (8.6)	35 (66.0)	9 (17.0)	9 (17.0)	0.01
I will do CPR if HIV/AIDS patients need it	70 (79.6)	16 (18.2)	2 (2.2)	33 (97.1)	1 (2.9)	0 (0.0)	18 (51.4)	15 (42.9)	2 (5.7)	0.04
Mean favorable attitude score (total possible score = 65)	53.40 ± 7.68 SD			53.86 ± 9.70 SD			53.09 ± 6.02 SD			0.64

^a^ Statements were reversely coded (i.e., strongly agree = 1, strongly disagree = 5).

**Table 5:** Anticipatory attitude (willingness) towards treating disadvantaged communities during CBDE.

Characteristic	Total N = 89 N (%)					Pre-CBDE Dental Students with Previous Community Experience Working with PLWH N = 36 N (%)					Pre-CBDE Dental Students without Previous Community Experience Working with PLWH N = 53 N (%)					*p*- Value
	Extremely Comfortable	Somewhat Comfortable	Neither Comfortable nor Uncomfortable	Somewhat Uncomfortable	Extremely Uncomfortable	Extremely Comfortable	Somewhat Comfortable	Neither Comfortable nor Uncomfortable	Uncomfortable	Extremely Uncomfortable	Extremely Comfortable	Somewhat Comfortable	Neither Comfortable nor Uncomfortable	Uncomfortable	Extremely Uncomfortable	
People with low income	52 (59.8)	26 (29.9)	9 (10.3)	0 (0.0)	0 (0.0)	25 (69.4)	8 (22.2)	3 (8.3)	0 (0.0)	0 (0.0)	27 (52.9)	18 (35.3)	6 (11.8)	0 (0.0)	0 (0.0)	0.37
PLWH	30 (34.9)	28 (32.6)	20 (23.3)	6 (7.0)	2 (2.3)	19 (52.8)	9 (25.0)	6 (16.7)	2 (5.5)	0 (0.0)	11 (22.0)	19 (38.0)	14 (28.0)	4 (8.0)	2 (4.0)	0.04
People who inject drugs	21 (24.4)	33 (38.4)	22 (25.6)	6 (7.0)	4 (4.6)	12 (33.3)	10 (27.8)	10 (27.8)	3 (8.3)	1 (2.8)	9 (18.0)	24 (46.0)	12 (24.0)	3 (6.0)	3 (6.0)	0.33
Homeless peolple	33 (37.0)	34 (38.2)	15 (16.9)	4 (4.5)	3 (3.4)	20 (55.6)	10 (27.7)	6 (16.7)	0 (0.0)	0 (0.0)	13 (24.5)	24 (45.3)	9 (16.9)	4 (7.6)	3 (5.7)	0.01
Mean favorable attitude score (total possible score = 20)	15.73 ± 4.06 SD					17.05 ± 2.8 SD					14.80 ± 4.5 SD					0.01

**Table 6:** Anticipatory attitude (willingness) towards treating disadvantaged communities after graduation.

Characteristic	Total, N = 89 N (%)		Pre-CBDE Dental Students with Previous Community Experience Working with PLWH N = 36 N (%)		Pre-CBDE Dental Students without Previous Community Experience Working with PLWH N = 53 N (%)		*p*-Value
	Yes	No	Yes	No	Yes	No	
People on a low income	88 (98.9)	1 (1.1)	35 (97.2)	1 (3.8)	53 (100.0)	0 (0.0)	0.78
Homeless people	87 (97.7)	2 (2.3)	35 (97.2)	1 (2.8)	52 (98.1)	1 (1.9)	0.22
People who inject drugs	82 (92.1)	7 (7.9)	31 (86.1)	5 (13.9)	51 (96.2)	2 (3.8)	.082
PLWH	85 (95.5)	4 (4.5)	34 (94.4)	2 (5.6)	51 (96.2)	2 (3.8)	0.69
Mean favorable attitude score (Total possible score = 4)	3.84 ± 0.58 SD		3.75 ± 0.77 SD		3.90 ± 0.40 SD		0.21

## Data Availability

The data that support the findings of this study are openly available in Havard Dataverse at https://dataverse.harvard.edu/dataset.xhtml?persistentId=doi:10.7910/DVN/N1BIR4 (accessed on 8 May 2024).
